# Extreme Concentration and Nanoscale Interaction of Light

**DOI:** 10.1021/acsphotonics.2c00187

**Published:** 2022-05-24

**Authors:** Gerd Leuchs, Alexey V. Andrianov, Elena A. Anashkina, Alina A. Manshina, Peter Banzer, Markus Sondermann

**Affiliations:** † Max Planck Institute for the Science of Light, 91058 Erlangen, Germany; ‡ Friedrich-Alexander-Universität Erlangen-Nürnberg, Department of Physics, 91058 Erlangen, Germany; § Institute of Applied Physics, Russian Academy of Sciences, 603950 Nizhny Novgorod, Russia; ∥ Lobachevsky State University of Nizhny Novgorod, 603022 Nizhny Novgorod, Russia; ⊥ Institute of Chemistry, St. Petersburg State University, 26 Universitetskii prospect, St. Petersburg 198504, Russia; # Institute of Physics, University of Graz, 8010 Graz, Austria

**Keywords:** focusing, light−matter interactions, quantum optics, nonlinear optics, field enhancement

## Abstract

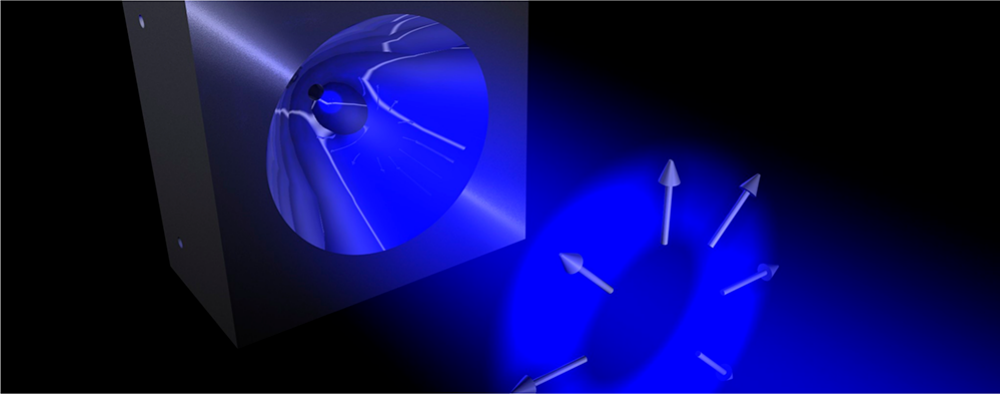

Concentrating light strongly calls for appropriate polarization patterns of the focused light beam and for up to a full 4π solid angle geometry. Focusing on the extreme requires efficient coupling to nanostructures of one kind or another via cylindrical vector beams having such patterns, the details of which depend on the geometry and property of the respective nanostructure. Cylindrical vector beams can not only be used to study a nanostructure, but also vice versa. Closely related is the discussion of topics such as the ultimate diffraction limit, a resonant field enhancement near nanoscopic absorbers, as well as speculations about nonresonant field enhancement, which, if it exists, might be relevant to pair production in vacuum. These cases do require further rigorous simulations and more decisive experiments. While there is a wide diversity of scenarios, there are also conceptually very different models offering helpful intuitive pictures despite this diversity.

## Overview

Optics is about the modes of the field, given by Maxwell’s equations, including boundary conditions imposed by matter and by the excitation of these modes described by quantum field theory and often visualized by Wigner functions in corresponding phase spaces. The parameters of the Wigner functions are conjugate pairs of field quadratures, and the modes are parametrized by the spatial mode pattern (including polarization) and the optical frequency or time, another conjugate pair. All these factors have to be considered when trying to understand optical phenomena. Here, we will address one of the most basic tasks in optics: the focusing of light, that is, concentrating the energy of the electromagnetic field as much as possible in one point in space or even in space–time.

One may say that the research on understanding the limits of concentrating light started more than 150 years ago. At that time, optical engineers and scientists were trying to understand the resolution limit of a microscope, which in a way is inverse to the problem of concentrating light. At that time, after a longer struggle, two alternative treatments accounted for the resolution limit of a microscope: one by Abbe^1^ and another one by Helmholtz^2^ and by Rayleigh.^3^ This diffraction limit still applies today, unless one images stochastic blinking molecules^4,5^ or molecules that can be switched using stimulated emission.^6^

While Abbe and also Helmholtz and Rayleigh discussed the inverse of focusing in the paraxial approximation with a scalar model for the light field, Richards and Wolf^7^ calculated the focusing for nonparaxial geometry, considering the full vector properties of the light field and for focusing from up to half the full solid angle, corresponding to a numerical aperture in air of unity. They found that a linearly polarized input beam produces a complex polarization structure in the focal area. Later, Bassett^8^ pointed out that, in addition to the plane wave basis, the multipole functions at every point in space also form a basis for mode functions. He argued that of all multipoles of the electromagnetic field, only the electric dipole wave contributes to the electric field at the focal point; note that this in reverse justifies Huygens’ principle and that for a given input power, the highest electric field strength is thus obtained by focusing an electric dipole wave. In this case, the polarization structure is complex at the input, but more homogeneous around the focus. The relevance of the polarization structure to tight focusing was recognized independently by Quabis et al.^9^ and by Youngworth and Brown.^10^ They showed that, in high numerical aperture focusing from 2π solid angle, cylindrical vector modes best match an ingoing dipole wave. Since this match is only partial, some polarization structure remains at the focus. Despite this polarization structure, such a tightly focused beam can be used to determine optical properties of small structures such as nanoparticles^11−13^ and flakes.^14^ Characterizing nanostructures requires full control of all parameters of the light field. The tighter spot obtained when focusing from close to 2π solid angle was demonstrated experimentally^15^ using radial polarization at the input, best matching an ingoing electric dipole wave when focused. For a review of the many uses of cylindrical vector modes see refs (16 and 17).

In this Perspective, we will start by discussing the relevance of the vectorial property of light for focusing and for coupling to nanostructures and will then give a more detailed account of concentrating light at one point in space or space-time sending in light from the full 4π solid angle in different scenarios: in free space, with a sub wavelength antenna such as an atom at the focal point, and in a homogeneous but optically nonlinear medium. We will refer to familiar observations, such as resonant field enhancement in the vicinity of a small antenna, discuss the apparent discrepancy between a diffraction limited focal spot and an ingoing dipole wave being “singular” at the origin, and speculate about a novel behavior when the focused light beam creates its own absorption through nonlinear interaction.

## Introduction to the Shape of Light: Paraxial and Nonparaxial Propagation Regimes

Light exhibits a number of different parameters, i.e., the wavelength, intensity, phase, polarization, and so on, which can be influenced, manipulated, or modified. Some of these parameters may also feature a spatial degree of freedom. In the following section, we discuss some selected examples of structured light with a strong emphasis on light beams.

### Structured Paraxial Light Beams

A fundamental Gaussian beam of light that, in contrast to a plane electromagnetic wave, features a position-dependent amplitude value in the beam cross-section, might be considered as the simplest example of a structured light beam, but light fields and beams can also be spatially tailored or sculpted in phase and polarization. In addition, also the temporal and spectral structure of light can be selectively controlled. The possibility to structure light fields opens up a rich plethora of different routes for both fundamental studies and applications.^18^

Besides the Gaussian beam mentioned above, many other solutions to the paraxial wave equation can be analytically derived. They are excellent approximations to the light beams utilized in the lab. Among others, Hermite–Gaussian and Laguerre–Gaussian (LG) modes represent full sets of scalar spatial modes featuring a spatially structured intensity and phase distribution, while still exhibiting a homogeneous polarization (e.g., linear). The phase structure also gives rise to other intriguing phenomena, such as orbital angular momentum, a direct consequence of helical phase fronts naturally appearing in subsets of LG modes.^19^ On the optical axis, these modes feature phase singularities, a phenomenon that is at the heart of singular optics. Probably the best known type of angular momentum light may carry is the spin, resulting from the polarization with the field vector spinning about the propagation direction for elliptically or circularly polarized light.^20^ Although spin angular momentum is a property connected to the local field and its dynamics, the polarization may in general also feature spatially nonhomogeneous distributions. In contrast to the above-mentioned singularities, there are also polarization singularities extending the area of singular optics. In fact, one subfield of polarization singularities deals with points of circular polarization or lines of linear polarization, where the intensity is not zero, embedded in an elliptically polarized environment.^17,21−23^

In another subfield, the intensity at the singular point must be zero. Simple, instructive, and prominent examples are paraxial vectorially structured cylindrical vector beams (CVB),^9,16,17^ in particular radially or azimuthally polarized modes. They feature a cylindrically symmetric field distribution and are often referred to as doughnut modes, owing to their ring-shaped intensity profile (similar to LG modes). Locally, they are linearly polarized in the paraxial regime of propagation, while the polarization direction is position-dependent and either oriented along the radial or azimuthal direction. CVBs find a wide range of applications, from metrology and sensing to optical communication and information transfer,^16−18,24−26^ as a direct consequence of spatial polarization, intensity, and phase distributions, as well as of their inherent structure and correlations. They also play a pivotal role in this Perspective. It goes without saying that the complexity of paraxial beam shaping can be further increased. An intriguing example is so-called Poincaré beams.^27^ They feature all possible 2D polarization states in their beam cross-section, covering the whole Poincaré sphere.

Over the last decades, many different methods for the generation of paraxial structured light (scalar and vectorial) have been introduced.^16−18^ In most free space approaches to beam shaping, that is, without any cavity, the desired polarization, phase, or intensity distribution or modal structure is imprinted on the incoming beam of light (usually a fundamental Gaussian mode) by a local position-dependent action. To manipulate the polarization or phase, for example, waveplates can be used, which are arranged such that their fast and slow axes exhibit an orientation dependent on the transverse coordinate with respect to the incoming beam. Conventional waveplates, micro- and nanostructures, or liquid crystal molecules are prominent examples acting as building blocks of beam-shapers, such as segmented wave plates,^15^ spatial light modulators,^28^ θ-cells and q-plates,^29−31^ and metasurfaces.^32−34^ All these operations convert the initial mode of the light field.^35,36^ In addition, beam shaping based on nonstructured, homogeneous, and isotropic media has also been discussed,^37−39^ taking advantage of, for instance, Fresnel coefficients, the Brewster angle, or other aspects related to geometrical optics.

### Field Engineering: Transverse and Longitudinal Fields by Spatial Confinement

Strictly speaking, the analytical and paraxial beam-like solutions mentioned above do not solve Maxwell’s equations or the full wave equation exactly. The simple reason for this is the fact that the underlying analytical expressions for many kinds of beams are a result of the paraxial approximation, assuming close to collimated propagation. Moreover, this step also restricts the electric and magnetic fields to oscillate in a transverse plane with respect to the propagation or optical axis, just like for a plane wave. It can be shown, however, that spatially confined fields usually feature also electric and magnetic field components oriented along the mean propagation direction, that is, the longitudinal direction, in addition to the transverse field components.^40^ It is worth noting here that this statement is true for all kinds of different fields and types of confinement, such as focusing or converging propagation,^7,9,10,15^ evanescent waves or near-fields, and fiber or waveguide modes. The contribution of longitudinal field components to the overall field strongly depends on the strength of the confinement. The actual three-dimensional field, for example, in the cross-section or focal plane of a focused light beam, will sensitively depend on the polarization, phase, and intensity distribution of the beam sent into the focusing system. Theoretically, such focal field distributions can be conveniently calculated using vectorial diffraction theory,^7^ which is essentially based on the decomposition of the input beam at the focusing lens or mirror into individual plane wave components eventually propagating to the focus where they interfere to form the focal spot.

If light is interacting with matter, as discussed in detail in the next section, also evanescent fields start to contribute to the field distributions. For the structure of near-fields, the geometry, dimensions, and material of the matter system a light field is interacting with, play a pivotal role. They constitute extra knobs waiting to be turned for selective spatial tailoring of electromagnetic fields, leading to precise control over near-field enhancements, hot-spot distributions and more. Especially when combined with sculpted excitation fields, many pathways open up (see next sections).

Experimentally, a plethora of different scalar and vectorial spatial modes of light have been demonstrated, implemented, and utilized. Especially at the nanoscale, that is, under strong spatial confinement conditions, exotic topological field structures have been proposed and experimentally shown, ranging from Möbius strips and ribbons formed by the polarization,^41−47^ knotted lines of phase singularities or polarization,^48,49^ skyrmions,^48^ and spatiotemporal vortices,^50−52^ to just name a few. The possibilities seem endless, with the only limitation being the requirement for the field modes to solve Maxwell’s equations.

This discussion emphasizes how many different ways confined fields can be selectively engineered by manipulating the input or excited light beams to be concentrated by focusing elements or interacting with material systems.

### Studying Nanosystems with Sculpted Fields

**Figure 1:**
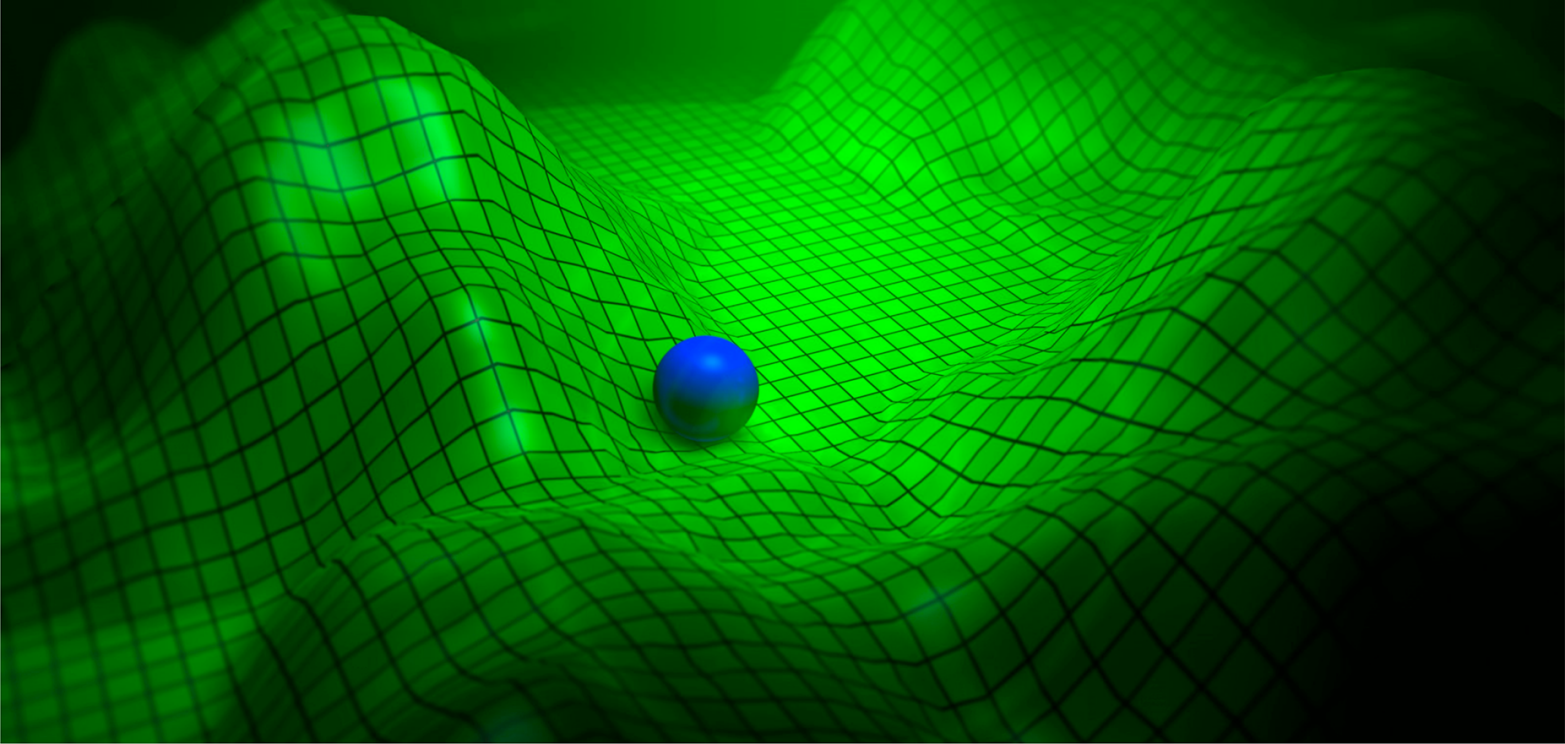
Artistic illustration of a nanoscopic object placed in a tailored electromagnetic field landscape changing at a subwavelength scale, showing the 2D spatial distribution of just one parameter. The interaction will strongly depend on the relative position, leading to multiple interaction schemes and enabling controllable field manipulation, beam steering, and so on. At the same time, information on the nanosystem itself can be retrieved (position, size, shape, material, etc.) via an in-detail analysis of the scattered light.

When light is interacting with matter, all the way from macroscopic via microscopic to nanoscopic length scales, this interaction will strongly depend on both the light field itself as well as the matter system. With the discussion above, it might not come as a surprise anymore that by sculpting parameters such as wavelength, amplitude, phase, and polarization, bespoke interaction scenarios can be realized (see Figure 1), while some of those parameters mutually depend on each other.

The various possibilities of sculpting the electromagnetic field at the subwavelength scale, as discussed above, open up countless pathways for studying the optical, geometrical, and morphological properties of nanoscopic systems. If placed in a tightly focused beam or electromagnetic near-field distribution, a nanostructure will interact with the light field in a strongly position-dependent manner. If the excitation field distribution is experimentally known,^53,54^ detailed information on the nanoscale matter system can be retrieved by analyzing the scattered light carefully,^11−13,55^ even though the studied object can be way smaller than the wavelength of light or the resolution limit of conventional microscopes. Also, in microscale systems, where conventional polarimetry or ellipsometry methods still fail, small focal spots and their rich features provide for powerful alternatives to study optical properties of a specimen.^56,57^

The most prominent example of structured light-based applications coming to mind are structured illumination microscopy (SIM)^58,59^ and stimulated emission depletion (STED).^6^ However, especially in the latter case, the rich substructure of spatially confined electro-magnetic fields is not fully exploited, because the underlying effect is mainly based on the electric field intensity distribution. Nonetheless, the last two decades have shown that by taking advantage of the subwavelength features in structured and confined fields, the range of applications can be drastically extended and existing applications can be optimized. The rich and intriguing features of all kinds of nanoscale fields, for example, related to longitudinal fields or optical angular momenta,^55,60,61^ have paved the way for bespoke techniques in the context of precise nanometrology,^62−64^ nanoscale traffic control for integrated photonics,^64−66^ advanced nanospectroscopy,^13,67−69^ excitation of dark modes or anapoles,^13,70−73^ and optical trapping.^74−79^ Furthermore, the developments in the field of extraordinary angular momenta of light,^55^ an inherent feature of confined fields, also set the stage for further developments in quantum optics.^61^

## Focusing from a Nearly Full 4π Solid Angle

**Figure 2:**
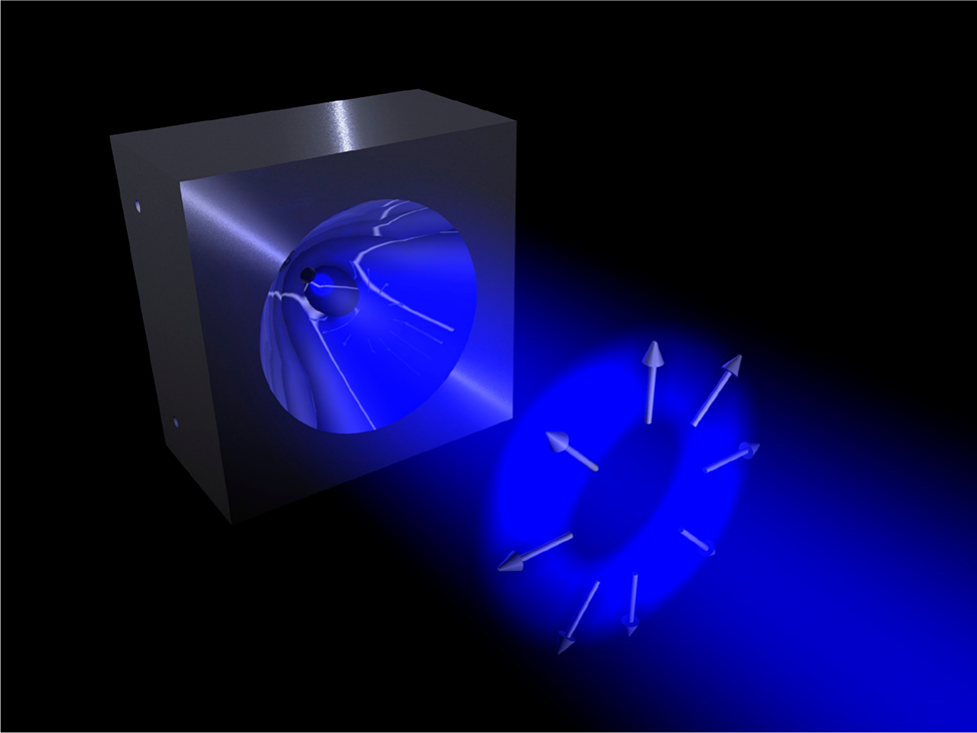
Pictorial view of a radially polarized cylindrical vector beam impinging onto a parabolic mirror. The parabolic mirror shown in the image has the same geometry as the mirrors used in refs (80 and 81).

Focusing light from a full 4π solid angle in the lab is not an easy task. The first possibility that may come to mind is the geometry used in 4π microscopy, using two opposing lenses. But even with the highest numerical aperture readily available (NA = 0.95), two such lenses together will focus only 69% of the full solid angle. In comparison, just as an example, a deep parabolic mirror (see Figure 2) with an opening angle of 46°, as in refs (80 and 81), covers 82% of the full solid angle. The parabolic mirror serves as a mode converter,^82^ for example, converting a paraxial radially polarized cylindrical vector mode into a good approximation of an ingoing electric-dipole wave. For this particular example, a parabolic mirror with the above geometry appears to be even more advantageous, since it covers 94% of the light power when weighting with the dipole’s intensity distribution.

### Diffraction Limited Focusing in Free Space

The expansion of the electromagnetic field into its multipole constituents as well as the consideration of time reversal of spontaneous emission (as in ref (9), see also below) suggest that, for the highest concentration, one should send in just an electric dipole wave, readily provided by focusing an appropriate cylindrical vector mode by a deep parabolic mirror. One technique to calculate the field distribution at the focus is to expand the dipole wave into a superposition of plane waves,^7^ which gives the familiar diffraction limited spot.

But how about letting the dipole wave propagate all the way to the origin where it starts to develop singular behavior? This is a bit more tricky, because the electromagnetic field part is by itself not a solution of Maxwell’s equations. It is a solution only in combination with an oscillating electric dipole. The scenario is even a bit unusual because an ingoing dipole wave without any losses will have to reappear as an outgoing dipole wave, forming a standing dipole wave, in contrast to focusing with a lens, where in- and outgoing beams do not interfere. In free space, that is, in the absence of any electric dipole, you can still use the electrical dipole solutions if the two electric dipoles, the one associated with the ingoing and the other one associated with the outgoing dipole wave cancel each other. Consequently, one has to combine the ingoing dipole field *E*_in_ and the outgoing dipole field *E*_out_ with a minus sign, resulting in the electric field *E*_standing_ of the following standing wave: *E*_standing_ = *E*_in_ – *E*_out_. An alternative interpretation of the minus sign may be that in this extreme focusing regime the Rayleigh length is zero and the 180° Gouy phase shift appears abruptly when the wave is passing through the focus.

**Figure 3:**
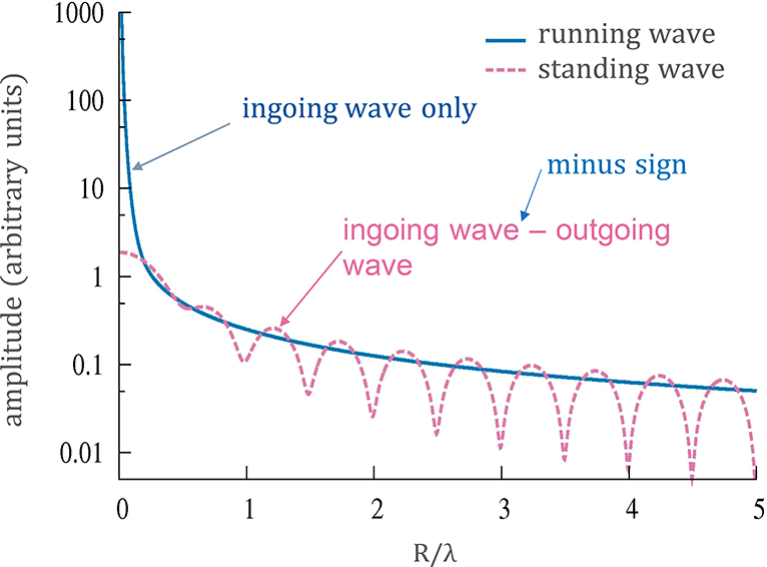
Comparison of (1) the radial dependence of the amplitude of an ingoing dipole wave (running wave) absorbed at the focus, *R* = 0, and (2) the difference of an ingoing and an outgoing dipole wave resulting in a standing dipole wave, both dipole waves having the same spherically integrated power in the far field.

As it turns out, this cancels all the singular behavior,^83,84^ resulting in a sinc-function-like spatial distribution.^85^Figure 3 shows the resulting standing dipole wave intensity, the envelope of which has a finite maximum at the focal point and a width corresponding to the diffraction limit. The singular behavior of just the ingoing dipole wave, absorbed by the subwavelength electric dipole at the focus is shown for comparison. We note that in the multipole expansion, as carried out, for example, in ref (8), the radial dependence of the field readily includes the standing-wave property.

**Figure 4:**
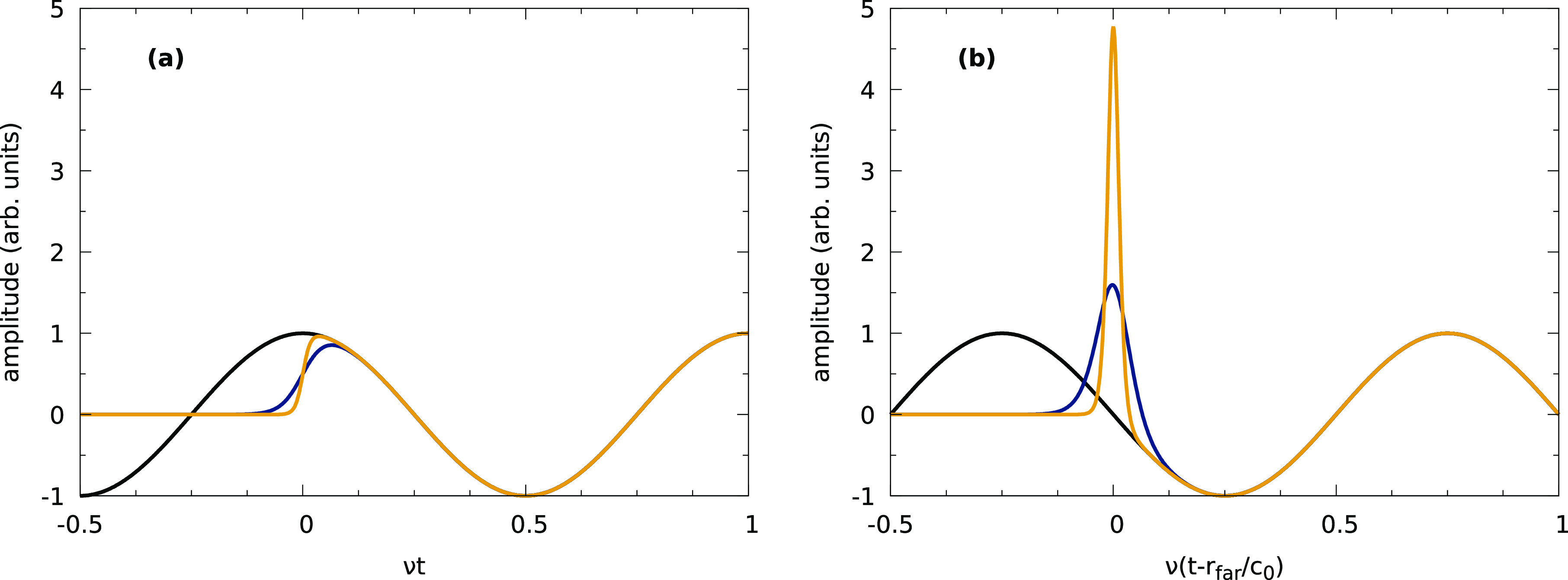
A transient “singular” behavior may be expected if the ingoing dipole wave is switched on with a sharp rising front. (a) The temporal evolution of a continuous sinusoidal wave with frequency ν and two wave fronts with different steepness levels at one point in space at distance *r*_far_ far away from the origin. (b) The modeling of the field distribution at the origin for times close to the moment when the front moves through the origin,^84^ clearly displaying transient enhancement. In both cases, the field amplitude is normalized to the continuous sinusoidal wave.

Next, one may wonder what happens if the incoming dipole wave is time-dependent, in particular if it is switched on abruptly, that is, faster than the steepest slope of the sinusoidal wave. In that case, there would only be an ingoing dipole wave up to the point in time when the sharp front moves through the focus. At this point, the singular behavior leads to some enhancement, which however will be transient, because as soon as the incoming front will have moved through the focus, there will be a standing wave in the focal region and the enhancement will disappear. Switching light waves on so quickly is difficult, and no experimental test was done, but this scenario was modeled theoretically (see Figure 4), confirming the above expectation,^84^ suggesting that a potential transient field enhancement is created by a sharp rising front of the incoming light beam.

Such transient peaks can also be understood by discussing the topic in the frequency domain instead of in the time domain. The sharp front is associated with transient higher frequency components, which will give rise to a sharper and hence higher amplitude transient peak at the moment when the front passes the focus (note that the intensity at the focus of an electric-dipole wave of wavelength λ is proportional to 1/λ^2^, see, for example, ref (8)).

### Focusing to a Single Sub Wavelength Resonant Antenna

While concentrating light in structures on the nanometer to micrometer scale is already a challenging task, concentrating light within the dimensions of a single atom seems to be the most extreme focusing problem. To this end, Paul and Fischer^86^ studied how a single subwavelength dipole antenna (such as an atom) distorts the initially parallel energy flux lines of an incident plane wave. It is remarkable to see how, in the region around the atom, the flux lines are distorted to match an ingoing dipole wave, which also results in a scattering cross section much larger than the physical dimensions of the atom (see also the introduction of ref (80)).

This indicates that focusing a suitably prepared dipole wave will be sufficient to concentrate the field energy within atomic dimensions. So, the question at hand is then whether a single photon with a suitably chosen mode function can bring the atom from its ground state to the excited state with 100% probability? From a theory perspective, the answer will clearly be affirmative.^87^

There are two equally valid approaches in deriving the recipe for such perfect coupling in free space. The first one is based on a time-reversal argument:^9,82,88−90^ If one would be able to monitor the temporal evolution of the spontaneous emission of a photon and revert this evolution in all degrees of freedom, the result would be as follows: A photon impinges onto an atom in the ground state, the absorption process will start, and at some point, the field energy will be completely transferred onto the atom, which is then in the excited state. Neglecting the degrees of freedom related to the motion of the atom,^91^ the decisive information is found in the spatiotemporal degrees of freedom of the emitted photon. For most of the atomic transitions of interest, the emitted photon has the spatial mode function of an electric dipole. Thus, when not reversing the evolution of an actually emitted photon (where imperfections are unavoidable due to fundamental constraints^90,92,93^), but rather focusing properly tailored light from whatever source onto an atom, the mode function of the focused field has to be the one of an electric dipole mode with its singular behavior when approaching the focus, while still being outside the atomic charge distribution.

The optimum mode shape for coupling to an atom in free space can also be derived from another, somewhat more formal approach. When targeting an electric-dipole transition with dipole moment μ⃗, the interaction between an atom and the electric field *E⃗* at the position of the atom is given by the interaction Hamiltonian −μ⃗·*E⃗*. Recalling that only the electric-dipole modes possess a finite electric field in the origin,^8,94^ the magnitude of the electric field seen by the atom is maximized by shaping the incident light to resemble the electric-dipole radiation matching the far field of the atomic dipole μ⃗.

Both of the above approaches do not only imply to shape the amplitude distribution and the local state of polarization of the focused light field. It is also mandatory to focus onto the atom from full solid angle in achieving perfect coupling in free space. Possible focusing setups for (close to) full solid angle illumination can be based on two microscope objectives of high NA (as used in 4π microscopy^95^) or deep parabolic mirrors with a depth much larger than their focal length.^82,88^ Deep parabolic mirrors have also been suggested to couple atoms to a squeezed vacuum^96^ or to collect the complete emission of an atom.^97,98^

One could speculate about other focusing geometries which might result in an extreme concentration of the light field, for example, by focusing more than a single beam. One such geometry is obviously found in the already mentioned 4π-microscopy.^95^ Furthermore, there are suggestions to boost the intensity per focused light power by focusing several suitably arranged laser beams each with optics of low numerical aperture.^99^ These approaches reconvene with the full-solid-angle approach when realizing that every focusing geometry can be mapped onto focusing from full solid angle in simply setting the field distribution to zero in regions from which no light is focused. Of course, this results in nonperfect overlap with the field distribution of a dipole wave. Nevertheless, the accompanying losses in focal intensity can be minimized by optimizing the geometry and may be acceptable for particular applications.

Experiments on focusing light onto atoms or other single quantum targets in free space have been performed with molecules,^100,101^ epitaxially grown semiconductor quantum dots,^102^ and neutral atoms,^103−105^ as well as ions.^80,106−110^ Focusing onto the atomic target from a large fraction of the full solid angle has been pursued with a deep parabolic mirror^80,110^ as well as with two lenses in a 4π microscopy scheme.^111^ The perfect excitation of a single atom with a single photon in free-space is still pending.

So far we discussed the concentration of light using dipole waves or superpositions thereof (model “dipole wave”). But there is a second model “antenna back-reaction”, which goes as follows: The perfect absorption of light by an atom can be seen as the destructive interference between the incoming light which is transmitted and the light re-emitted by the atom. The far field of the re-emitted dipole radiation interferes destructively with the excitation light, while the nonpropagating near-field part of the re-emitted light persists, concentrating the energy near the atom^86^ (see also Zumofen et al.^112^ for a similar argumentation on atomic dipoles reflecting light). The excitation of local, nonpropagating near-field components and the associated field enhancement are also found when focusing light onto plasmonic nanoparticles.^113−116^ When the plasmonic particle is designed properly, one should be able to observe full resonant absorption of the whole incoming field,^117^ similar to when focusing onto a quantum absorber. Such particles suppress the outgoing part of the focused field and lead to a singular-like behavior in the vicinity of the particle, that is, the particle leads to field enhancement.

#### Concentrating Light in a Resonator

There is an interesting analogy between the dynamics of light pulses coupling to an optical resonator and of coupling a single photon to a two level atom.^118^ Using again the time reversal symmetry argument, the pulse that couples perfectly to a Fabry–Perot interferometer with only one input–output coupling mirror is a growing exponential with the growth rate adjusted to the decay constant of the cavity. This was experimentally demonstrated for exponentially shaped coherent states^119^ and for an asymmetric single photon pulse, both with a rising exponentially leading edge and a sharp cutoff on the trailing edge.^120^ The smallest linear resonator supporting only one mode for a particular wavelength will have a cavity length of half this wavelength. Going to 3D, a spherical cavity supporting only one mode will have a volume of about (λ/2)^3^, which roughly corresponds to the diffraction limited focal volume when focusing in free space. Again, the mode coupling most efficiently to such a spherical resonator will be an ingoing dipole wave. For the same incoming power, the cavity will build up the energy stored inside! But this is not the limit: if the real part of the dielectric function inside the resonator is zero, all frequencies will be resonant, because the optical phase inside will be the same everywhere, as in the case of a waveguide.^121^ Taking this to the extreme, one ends up at an atom: on resonance, the atom will scatter photons with 90° phase shift corresponding to the zero real part of the “dielectric function” of the atom. While there are all these close analogies, the difference is that in a resonator the energy stored inside is light energy, while the atom goes to the excited state and the stored energy is the potential and kinetic energy of the excited electron.

### Focusing in a Homogeneous Nonlinear Medium

Finally, one might speculate about the existence of so far unobserved effects in nonlinear homogeneous media when using a 4π focusing geometry. As already discussed above, the diffraction limited intensity distribution in the focal region can be understood as a superposition of an inward and an outward moving dipole wave.^83−85^

In the inhomogeneous case, this superposition can be disturbed by, for example, either absorption from a quasi-resonant nanoparticle at focus^117^ or by a particle that alters the relative phase of in- and outward propagating solutions.^122^ Either mechanism results in an enhancement of the field amplitude beyond the diffraction limit. But how about the homogeneous nonlinear medium? The absence of a sink for the electromagnetic field would let us expect that there is no such enhancement (at least when operating far from the resonance of a gaseous medium as in ref (81)). But what will happen when the field pumping the nonlinear process is strong enough to convert pump photons to photons at harmonic frequencies? Does this constitute some sort of “self-induced” absorbing structure of subwavelength dimensions (see next section)? If yes, will the loss of pump photons suffice to result in a recognizable, that is, measurable rise of the pump field amplitude in the vicinity of this self-induced absorber? What is clear so far is that answering these questions in an experiment demands for a setup that is as close to the ideal scenario as possible. This test is still pending.

For high enough laser intensities the vacuum is also a homogeneous nonlinear medium.^123,124^ The required laser intensities have been estimated^125^ but not yet reached in the experiment. Such experiments are envisioned as part of the Extreme Light Infrastructure Project ELI^126^ and the Exawatt Centre for Extreme Light Studies XCELS,^127^ and concentrating light efficiently might play a helpful role.^128^ If the above speculations were indeed to lead to some type of field enhancement, then this would also be relevant for determining the required laser threshold intensity for pair creation.

## Nonlinear Light–Matter Interactions Beyond the Paraxial Regime

The huge majority of experiments in nonlinear optics has been conducted in the paraxial regime (see ref (81) for hints to some exceptions). In textbooks, either plane waves or approaches based on the paraxial wave equation are typically considered to describe the physics of nonlinear optical phenomena.^129^ One might thus be mislead to think that nonlinear optics would be out of the scope of a discussion on concentrating light to the utmost limits. On the contrary, focusing one (or maybe even several) of the light beams participating in a nonlinear optical process such that the light is concentrated to subwavelength dimensions opens the opportunity to “rethink” several aspects of nonlinear optics. A first step in this direction was done recently in investigating the process of third-harmonic generation in a homogeneous medium, such as a noble gas, when focusing the pump light from full solid angle.^81^

**Figure 5:**
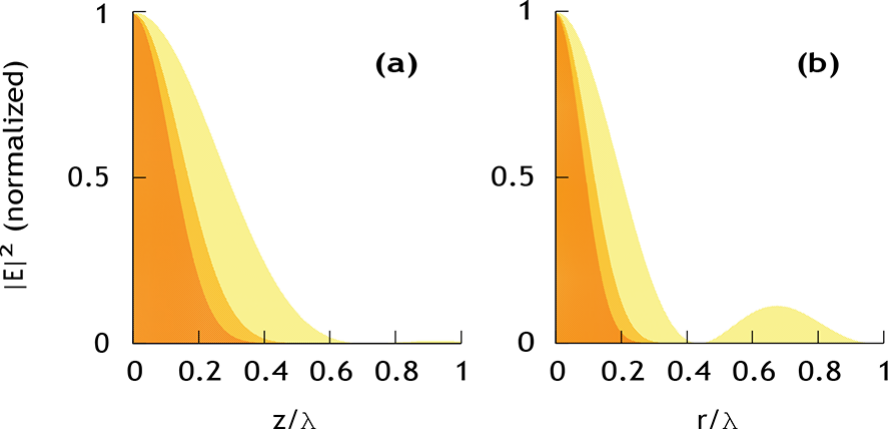
Simulated intensity distribution along the optical axis (a) and in radial direction in the focal plane (b) when focusing a linear electric dipole wave from full solid angle with a parabolic mirror. The radiation pattern of the focused light field resembles the one of an electric dipole oscillating along the optical axis. The shaded regions denote the focal intensity distribution and its third and fifth power (from bright to dark color).

In the generation of harmonics, the relevant quantity driving the nonlinear process is the intensity distribution of the pump light to the power of *n*, where *n* is given by the mechanism underlying the wave mixing. Thus, the already tight focal distribution obtained in a 4π focusing geometry is effectively narrowed, meaning that the nonlinear process is only driven effectively in a region much smaller than the diffraction limited focal volume (see Figure 5). Thus, the nonlinear interaction practically occurs only in a tiny region with a diameter of order λ/10. It is more than intuitive that on such a short spatial scale a considerable dephasing between pump light and frequency converted light cannot occur. So one might ask whether there are qualitative changes in the nonlinear optical interaction between the paraxial regime and the extreme full solid angle converging geometry. First investigations have shown on the one hand that the phase matching condition is strongly modified and on the other hand that essential properties of third-harmonic generation are retained in a 4π geometry,^81^ rendering momentum conservation a common decisive constraint in either regime. More detailed investigations are under way.

## Conclusions and Perspectives

Paraxial (cylindrical) vector beams and their focused nonparaxial 3D counterparts provide a plethora of intriguing phenomena and opportunities at the foundation of nanophotonics. This ranges from studies of the spatial degrees of freedom of light, the longitudinal and transverse aspects of the full 3D electromagnetic fields upon confinement, selective excitation of nano- and subnanosystems all the way to the limits of concentrating light in empty space. The latter is complicated by the vacuum ultimately being an optical nonlinear medium. For visualizing and simulating the concentration of light, different models have been used in the literature, the one explicitly considering the back reaction of electrical currents to the field (antenna back-reaction model) and the other one where source-field combinations such as an oscillating electric dipole and the corresponding dipole wave are used as the basic building blocks (dipole wave model). It is no surprise that in the standard scenarios both models predict a similar behavior. However, as discussed, there are challenging scenarios, not yet explored, neither experimentally nor by a rigorous theoretical study, and where surprises may be waiting and where one model may be more helpful than the other one for developing an intuitive understanding. This concerns situations involving nanostructures displaying nonradiative losses or a homogeneous medium with nonlinearity-induced losses, the latter with a potential impact on pair generation in the vacuum with intense laser light. Lastly, the ability to explore the full 3D field opens possibilities for nonlinear optics under conditions very different from standard paraxial nonlinear optics.
